# Biogenesis and Dynamics of Mitochondria during the Cell Cycle: Significance of 3′UTRs

**DOI:** 10.1371/journal.pone.0000107

**Published:** 2006-12-20

**Authors:** Marta Martínez-Diez, Gema Santamaría, Álvaro D. Ortega, José M. Cuezva

**Affiliations:** Departamento de Biología Molecular, Centro de Biología Molecular Severo Ochoa, Universidad Autónoma de Madrid, Madrid, Spain; Wellcome Trust Sanger Institute, United Kingdom

## Abstract

Nowadays, we are facing a renaissance of mitochondria in cancer biology. However, our knowledge of the basic cell biology and on the timing and mechanisms that control the biosynthesis of mitochondrial constituents during progression through the cell cycle of mammalian cells remain largely unknown. Herein, we document the *in vivo* changes on mitochondrial morphology and dynamics that accompany cellular mitosis, and illustrate the following key points of the biogenesis of mitochondria during progression of liver cells through the cycle: (i) the replication of nuclear and mitochondrial genomes is synchronized during cellular proliferation, (ii) the accretion of OXPHOS proteins is asynchronously regulated during proliferation being the synthesis of β-F1-ATPase and Hsp60 carried out also at G2/M and, (iii) the biosynthesis of cardiolipin is achieved during the S phase, although full development of the mitochondrial membrane potential (ΔΨm) is attained at G2/M. Furthermore, we demonstrate using reporter constructs that the mechanism regulating the accretion of β-F1-ATPase during cellular proliferation is controlled at the level of mRNA translation by the 3′UTR of the transcript. The 3′UTR-driven synthesis of the protein at G2/M is essential for conferring to the daughter cells the original phenotype of the parental cell. Our findings suggest that alterations on this process may promote deregulated β-F1-ATPase expression in human cancer.

## Introduction

Cellular proliferation is an energy consuming activity that is stringently controlled by checkpoints of the cell cycle [1]. Transition from one phase of the cycle to the next is coordinated by the expression of specific cyclins and the sequential activation and inactivation of cyclin-dependent protein kinases [2]. Uncontrolled proliferation is one of the hallmarks of the cancer cell [3] that most often results from genetic alterations and/or the inactivation of master regulators of the cell cycle [4]. Cells that make the decision to divide must be therefore metabolically prepared to deal with the energetic demand imposed by proliferation. Alternatively, the cells can become reversibly arrested at the G1/S boundary (restriction point) of the cell cycle. In fact, compromising the cellular ATP levels by inhibition of mitochondrial oxidative phosphorylation [5], [6] or by limiting the availability of glucose [1] or by genetic alterations that compromise the bioenergetic activity of mitochondria [7] result in G1 arrest of the cells. The G1/S arrest is triggered by a metabolic stress checkpoint of the cycle that is controlled by the activation of AMP-activated protein kinase (AMPK) [1] which is a metabolic sensor of the energy charge in higher eukaryotic cells [8]. The activation of AMPK promotes the phosphorylation of p53 at Ser15 [1], a modification that prevents its degradation and results in the cellular accumulation of p53 and cell-cycle arrest. Various studies have shown that entry of cells into the G1 phase of the cycle is associated with a burst of mitochondrial activity [5], [9]. However, it appears that progression through the cycle is supported by non-respiratory modes of energy generation [10]–[12]. In fact, very recent findings in cells of mammals indicate that cyclin D1 which is involved in the phosphorylation and inactivation of the retinoblastoma protein, marking the entry of cells into the S phase of the cycle, inhibits mitochondrial function [13] and represses the activity of NRF-1 [14], a nuclear factor that masters the transcriptional expression of nuclear-encoded mitochondrial genes [15].

Mitochondria participate in a large number of essential cellular functions. Genetic or epigenetic alterations that impact on mitochondrial functions are thus involved in the development of human pathologies with quite different phenotypic presentations [16], that include physiological ageing [17]. The provision of metabolic energy by oxidative phosphorylation (OXPHOS) is the best characterized function of mitochondria. In the process of oxidative phosphorylation, ATP is synthesized from ADP and Pi by the mitochondrial H^+^-ATP synthase [18], a rotatory engine complex of the inner mitochondrial membrane that utilizes as driving force the proton electrochemical gradient generated by the respiratory chain [19]. The catalytic activity of the H^+^-ATP synthase is located in the β-subunit of the water-soluble F1 portion (β-F1-ATPase) of the complex which is encoded in the nuclear genome [20]. The regulation of the expression of β-F1-ATPase is exerted at the level of translation [21]–[25]. The β-F1-ATPase mRNA (β-mRNA) further provides an example of a mitochondria-localized mRNA in both mammalian [26]–[29] and lower eukaryotic cells [30], [31] whose efficient translation depends on the 3′ non-translated region (3′UTR) of the mRNA [24], [30], [32], [33]. Translation masking of β-mRNA occurred both in the fetal liver [24] and in hepatomas [25], compromising the biogenesis of mitochondria during development and in oncogenesis [34], [35].

The biogenesis of mitochondria is a complex cellular event requiring the concerted expression of two physically separated genomes [15], [35]–[37]. However, and despite the relevant role played by mitochondria in the onset and progression of human pathology, our knowledge of the timing of the biosynthesis of the different mitochondrial constituents and on the mechanisms that regulate their biosynthesis during cellular proliferation are scarce or remain largely unknown. Even less explored are the dynamics and changes in mitochondrial morphology during mitosis, a process that is likely to impact on the development of mitochondrial function and on the segregation of the organelles during progression through the cell cycle. Because of the renaissance of mitochondrial studies in cancer biology [38]–[41], in this work we have studied the timing of the biosynthesis of different mitochondrial constituents during progression through the cell cycle and the changes on mitochondrial morphology and dynamics that accompany cellular mitosis. Mechanistically, we document the relevance that the 3′UTR (3′ non-translated region) of β-mRNA has for the synthesis of the protein at G2/M and illustrate the role that a regulatory mRNA sequence has for the appropriate biogenesis of mitochondria in the daughter cells with the same bioenergetic phenotype than that of the parental cell. This discovery highlights a previously unappreciated relationship between the control of translation at G2/M and the biogenesis of mammalian mitochondria that is likely to influence the cancer field.

## Results

### Biosynthesis of mitochondrial constituents during the cell cycle

**Figure 1:**
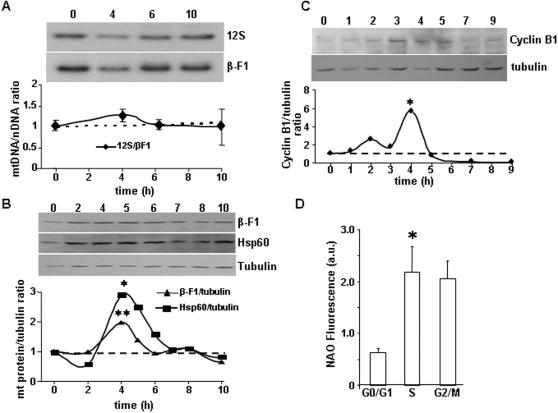
Changes in mitochondrial constituents during the cell cycle. Synchronized C9 cells (**A–C**) were recovered at the indicated time intervals and the relative cellular content of mtDNA (**A**) and proteins (**B–C**) determined. No significant changes in the cellular abundance of the mitochondrial 12S gene are observed. However, the cellular content of β-F1-ATPase, Hsp60 and cyclin B1 showed a significant increase at 4 h after release from the metabolic arrest. Representative experiments are shown (**A–C**). * and **, P<0.05 and <0.005 when compared with 0–2 h. **D**, The fluorescence intensity of the NAO probe was used to determine changes on mitochondrial mass during the cell cycle of non-synchronized C9 cells. The results shown are the means±SEM of three experiments. *, P<0.05 when compared with cells in G0/G1.

Subconfluent cultures of liver C9 cells were synchronized by metabolic arrest at the beginning of the S phase (see Fig. S1 in Supporting Information). The arrest of the cell cycle promoted a significant increase in the proportion of cells in G0/G1 concurrent with a reduction of the proportion of cells in S and G2/M (Fig. S1). At various times (0–10 h) after release from the metabolic block cells were recovered and the relative cellular content of mtDNA (Fig. 1A) and mitochondrial proteins (Fig. 1B) determined. It should be noted that at 4h after release from the arrest the percentage of cells in the different phases of the cycle were basically the same (Fig. S1) being the percentage of cells in G2/M 2-fold and 5-fold higher than that of non-arrested and arrested cells, respectively (Fig. S1). The relative cellular content of mitochondrial DNA (mtDNA), as determined by the ratio of the mitochondrial 12S rRNA gene to the nuclear β-F1-ATPase gene did not show significant deviations from the initially observed value (dotted line in Fig. 1A), suggesting that synthesis of nuclear and mitochondrial DNA is coordinated during cell cycle progression. Contrary to this finding, we observed that the relative cellular content of the catalytic subunit of the mitochondrial H^+^-ATP synthase (β-F1-ATPase/tubulin ratio) and of the structural heat shock protein 60 (hsp60) (hsp60/tubulin ratio) showed a significant increase at 4 h (Fig. 1B). Analysis of the cellular expression level of cyclin B1 (cyclin B1/tubulin ratio), that is known to peak at mitosis, also revealed a significant increase 4h after initiation of the cell cycle (Fig. 1C), suggesting that mitosis is the cell-cycle phase where the preferential synthesis of these mitochondrial proteins is taking place.

To assess the build up of the inner mitochondrial membrane during progression through the cycle changes in the amount of cardiolipin were determined by flow cytometry using the fluorescent NAO probe (Fig. 1D). The mean fluorescence intensity of NAO-stained cells increased significantly in S phase when compared to cells in G0/G1 (Fig. 1D). No further significant changes were observed in NAO fluorescence in the cells at G2/M (Fig. 1D), indicating that the biosynthesis of the phospholipid of the inner membrane is completed in S phase.

### Asynchronous accretion of mitochondrial proteins during the cycle

**Figure 2:**
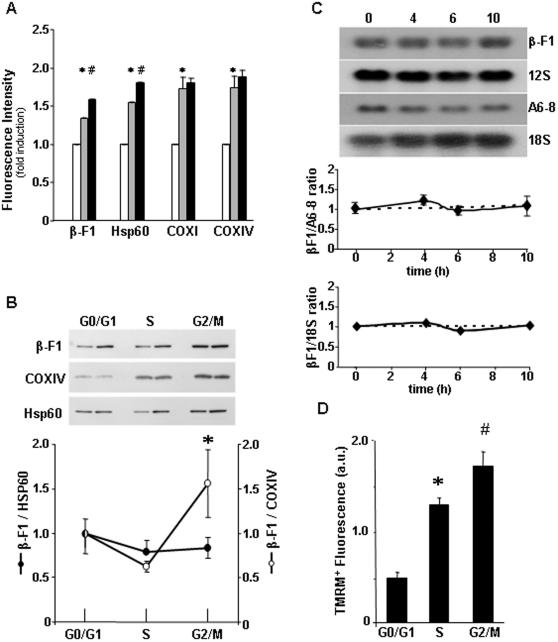
Asynchronous accumulation of mitochondrial proteins and development *ΔΨ*m during the cell cycle. **A**, Determination of the expression level of β-F1-ATPase (β-F1), Hsp60 and cytochrome c oxidase subunits I (COXI) and IV (COXIV) in C9 cells in the different phases of cell cycle by flow cytometry. Open bars, G0/G1; grey bars, S and black bars, G2/M. The results shown are the means±SEM of four experiments. * and ^#^, P< 0.05 when compared with cells in G0/G1 and S, respectively. **B**, C9 cells were sorted by flow cytometry according to their DNA content and the protein extracts fractionated and blotted with the indicated antibodies. The two bands shown under each condition are from two independent experiments. The results shown in the graph are the means±SEM of three experiments. *, P<0.05 when compared with cells in S phase. **C**, Synchronized C9 cells were recovered at the indicated time intervals and the relative cellular content of β-F1-ATPase mRNA determined by Northern-blot analysis. The mitochondrial encoded ATPase 6–8 mRNA and 12S rRNA and the nuclear encoded 18S rRNA were also determined. The results shown are the means±SEM of four experiments. **D**, FACS determination of the mitochondrial membrane potential (*ΔΨ*m) in C9 cells in the different phases of the cell cycle. The results shown are the means±SEM of three experiments. *, P<0.05 when compared with cells in G0/G1.

The cellular content of various mitochondrial proteins was further analyzed by flow cytometry in non-synchronized C9 cells (see Fig. S2 in Supplementary Information for controls of cytometry and representative experiments for each of the protein markers assayed). We observed that whereas the nuclear (COXIV) and mitochondrial (COXI) encoded subunits of respiratory complex IV (cytochrome c oxidase subunits IV and I, respectively) accumulated almost entirely during S phase (Figs. 2A and S2), the accumulation of β-F1-ATPase and hsp60 occurred throughout the cycle with a significant increase at G2/M when compared to cells in S phase (Figs. 2A and S2). The preferential build up of β-F1-ATPase and hsp60 at G2/M, and parallel accretion of these two proteins during the cycle, was further confirmed by western blotting of cells sorted according to their DNA content as revealed by the sharp increase in β-F1/COXIV ratio at G2/M (Fig. 2B) and the lack of significant deviations in β-F1/hsp60 ratio (Fig. 2B), respectively.

### The preferential synthesis of β-F1-ATPase at G2/M occurs in the absence of changes in mRNA abundance

To explore the possibility that β-F1-ATPase mRNA abundance could be regulated during the cycle gearing the synthesis and accumulation of the protein we determined the relative cellular content of β-mRNA in synchronized C9 cells at various times after release from the metabolic block. The expression of β-mRNA was determined (Fig. 2C) in the same cultures where the protein expression was assessed (Fig. 1B). No significant changes in the relative cellular content of β-mRNA (as assessed by any of the following ratios: β-mRNA/18S rRNA, β-mRNA/ATPase 6–8 mRNA and β-mRNA/12S rRNA) was observed (Fig. 2C). Furthermore, we also determined the relative expression of β-mRNA in cells sorted according to their DNA content and found the lack of significant changes in the normalized expression of β-mRNA in the different phases of the cycle (1.00±0.10, 0.83±0.12 and 1.21±0.10 for cells in G0/G1, S and G2/M, respectively). Overall, the results suggest that the accumulation of the protein during cellular proliferation (Figs. 1B, 2A and 2B) is mainly controlled at the level of translation although it cannot be excluded that mechanisms that control the subcellular localization of the mRNA [26]–[29], [31] might as well contribute to the observed accretion of the protein during the cell cycle.

### Functional differentiation of mitochondria during the cell cycle

To assess the stage of functional differentiation of mitochondria during the cell cycle, changes in the mitochondrial membrane potential (*ΔΨ*m) were determined by FACS analysis using the TMRM^+^ probe. The mean fluorescence intensity of TMRM-stained cells increased significantly in S phase when compared to cells in G0/G1 (Fig. 2D). However, and contrary to the results observed with the NAO probe (Fig. 1D), the mean fluorescence intensity of TMRM-stained cells further showed a significant increase at G2/M (Fig. 2D), indicating that full development of *ΔΨ*m is attained only after G2/M-dependent events have been completed.

### The presequence of β-F1-ATPase precursor targets gfp to the mitochondria

**Figure 3:**
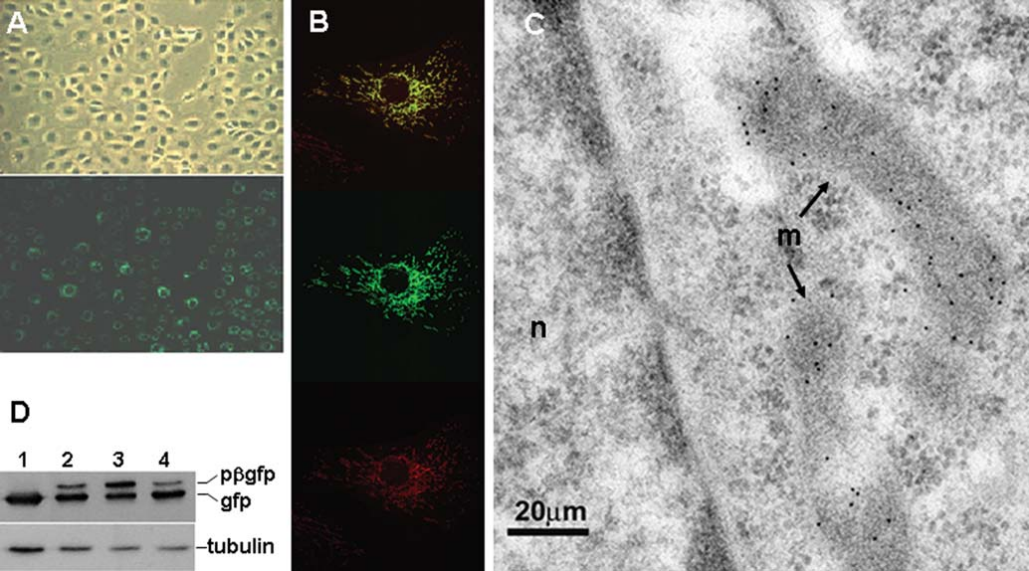
The presequence of β-F1-ATPase precursor targets gfp to mitochondria. The expression of gfp in C9 (**A,B)** and BHK (**C)** cells was assessed by fluorescence (**A**), immunofluorescence (**B**) and immunoelectron (**C**) microscopy. **A,** Illustrates by phase contrast (upper panel) and immunofluorescence (lower panel) the same low magnification field of C9-pβGFP3′β cells. **B,** C9-pβGFP3′β cells analyzed by immunofluorescence microscopy using anti-β-F1-ATPase antibody at 63× magnification. Upper panel, green gfp fluorescence; middle panel, red β-F1-ATPase immunostaining; lower panel, yellow merged image. **C,** Specific immunogold labeling (10 nm gold) of BHK mitochondria (m). Note the lack of gold labeling of other structures in the cytoplasm or in the nucleus (n) of the cell. **D**, Western blots of C9 (lane 1) and BHK cells (lanes 2–4) transfected with the pβGFP3′β construct. Fractionated proteins from the cellular extracts were probed with anti-gfp and anti-tubulin, the later as loading control. The migration of the pβ-gfp chimera is also indicated. In lane 3, BHK cells have been previously treated with FCCP (4 µM) plus oligomycin (2 µM) for 1 hour. Note the accumulation of pβ-gfp. In lane 4, BHK cells treated as in lane 3 were washed for 40 minutes before fractionation. Note the processing of pβ-gfp to mature gfp.

To test the possibility that changes in the translation efficiency of β-mRNA could trigger the accumulation of the protein during progression through the cycle we studied the expression of a gfp reporter derived from a construct that contained the 3′UTR regulatory element of β-mRNA translation [24], [32]. However, and because of the cytotoxic effect of gfp when it is expressed in the cytoplasm [33] the development of stable cellular clones by this approach resulted unsuccessful. Therefore, we decided to target gfp to the mitochondria by inframe fusion at the N-terminal of gfp the presequence of the rat liver β-F1-ATPase precursor protein. This strategy allowed the generation of stable clones of mammalian cells that express gfp in their mitochondria (Fig. 3A) as assessed by confocal (Fig. 3B) and immunoelectron (Fig. 3C) microscopy. Interestingly, it was not possible to detect significant amounts of the pβ-gfp chimeric protein in cellular extracts of the C9-pβGFP3′β clone (see Fig. 3D, lane 1), suggesting a fast and efficient *in vivo* import of the chimeric protein in this cell line. However, when transfecting the same construct into BHK cells we detected the pβ-gfp precursor (Fig. 3D, lane 2). Moreover, treatment of these cells with carbonyl cyanide p-trifluoromethoxy-phenylhydrazone (FCCP), a drug that collapses ΔΨm and prevents the import of precursor proteins into the organelle, revealed the rapid accumulation of pβ-gfp in BHK cellular extracts (Fig. 3D, lanes 3). Upon removal of FCCP from the culture medium, the pβ-gfp precursor was rapidly imported into mitochondria and processed to mature gfp (Fig. 3D, lane 4). These results illustrate cell-type specific differences in the import pathway of proteins into mitochondria in cells of mammals.

### Gfp derived from the C9-pβGFP3′β clone is preferentially synthesized at G2/M

**Figure 4:**
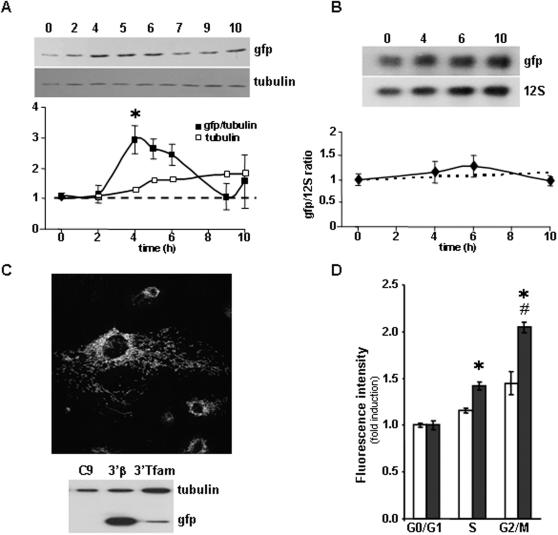
The 3′UTR of β-F1-ATPase mRNA controls the accumulation of the protein at G2/M. Synchronized C9-pβGFP3′β cells (**A,B**) were recovered at the indicated time intervals and the relative cellular content of gfp protein (gfp/tubulin ratio) (**A**) and gfp mRNA (gfp/12S ratio) (**B**) determined. The results shown are the means±SEM of three (**A**) and four (**B**) experiments. A significant accumulation of gfp protein was observed at four h after release from the metabolic arrest. *, P<0.0005 when compared with 0–2 h. No significant changes in the cellular abundance of gfp mRNA was observed during cellular proliferation. **C,** The upper panel shows a merged image of the immunofluorescence microscopy of C9-pβGFPTfam cells that illustrates the co-localization of gfp and β-F1-ATPase, thus revealing the efficient targeting of gfp to mitochondria in this cellular clone (for other details see Fig. 3B). The lower panel shows the western blots of fractionated proteins from cellular extracts of parental C9 (C9), C9-pβGFP3′β (3′β) and C9-pβGFPTfam (Tfam) cells simultaneously probed with anti-gfp and anti-tubulin. **D**, Analysis of gfp expression in C9-pβGFP3′β (closed bars) and C9-pβGFP3′Tfam (open bars) cells in the different phases of the cell cycle by flow cytometry. The results shown are the means±SEM of four experiments. *, P<0.05 when compared with C9-pβGFP3′β cells and ^#^, P<0.05 when compared S with G2/M in C9-pβGFP3′β cells.

Analysis of the relative cellular expression level of gfp in non-synchronized C9-pβGFP3′β cells, as assessed by the gfp/tubulin ratio, revealed a constant expression level of the protein as cells proliferate (data not shown). In contrast, the same analysis in synchronized C9-pβGFP3′β cells revealed a significant and preferential accumulation of gfp at 4 h after release from the metabolic block (Fig. 4A), indicating that the synthesis of gfp driven from the construct that contains the presequence and 3′UTR of β-mRNA coincides with that of the endogenous β-F1-ATPase (Fig. 1B) and is preferential at the time of mitosis (Fig. 1C). Determination of the relative cellular content of the chimeric gfp-mRNA in synchronized C9-pβGFP3′β cells revealed a constant expression level of this mRNA during the cycle (Fig. 4B), suggesting that the accumulation of gfp at G2/M is controlled at the level of translation.

### The 3′UTR of β-mRNA controls the accumulation of the protein at G2/M

In order to define which of the two β-mRNA elements (presequence and 3′UTR) is responsible for the preferential synthesis of the protein at G2/M we generated additional stable clones of the C9 cell line in which the 3′UTR of β-mRNA was replaced by the 3′UTR of mitochondrial transcription factor A (Tfam). The 3′UTR of Tfam lacks translation enhancing activity when compared to the activity of the 3′UTRs of other OXPHOS transcripts [33]. Consistent with this, we observed that the expression of gfp in C9-pβGFP3′Tfam cells was much less than that in C9-pβGFP3′β cells (Fig. 4C). However, the C9-pβGFP3′Tfam clone also efficiently targeted gfp to mitochondria in C9 cells showing no cellular accumulation of the pβ-gfp chimera (Fig. 4C, and data not shown).

To assess the role of the 3′UTRs in the synthesis of gfp during the cycle we determined the relative changes in gfp fluorescence of the C9-pβGFP3′β and C9-pβGFP3′Tfam cells (Fig. 4D). In both clones the mean fluorescence intensity of the cells increased as they progress through the cycle (Fig. 4D). However, the relative increase in cellular fluorescence observed in C9-pβGFP3′β cells exceeded by far that determined in C9-pβGFP3′Tfam cells both in S phase and at G2/M (Fig. 4D). In fact, C9-pβGFP3′Tfam cells did not synthesize the amount of gfp that is required for appropriate cellular division into daughter cells with the same maternal gfp phenotype (Fig. 4D). Therefore, the results support that the 3′UTR of β-mRNA is the core controlling element required for the biosynthesis of β-F1-ATPase and for the appropriate biogenesis of mitochondria during cellular proliferation.

### Dynamics of the mitochondrial network during mitosis

**Figure 5:**
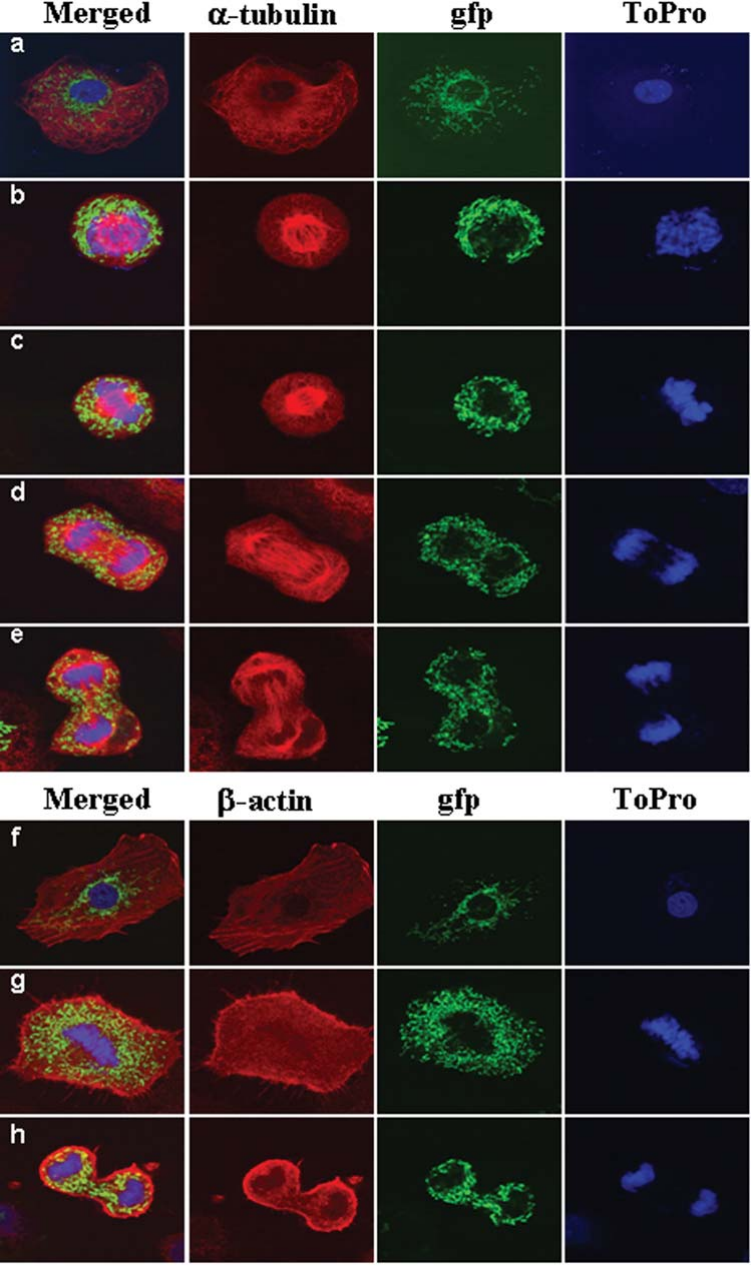
Morphological changes in the cellular mitochondrial network during mitosis. Synchronized mitochondria-tagged C9-pβGFP3′β cells were analyzed by immunofluorescence microscopy at four hours after release from the metabolic block. Typical morphologies of cells in interphase (a,f), prophase (b), metaphase (c,g), anaphase (d) and telophase (e,h) are shown at 60× magnification. The red fluorescence identifies the cytoskeletal proteins α-tubulin (a–e) and β-actin (f–h). The green fluorescence identifies mitochondria. The blue fluorescence reveals the stained nuclear DNA with the To-Pro probe.

The mitochondrial network is a dynamic structure that is permanently subjected to remodeling by changes in the morphology and subcellular localization of the organelles [42]. Organelle changes are relevant for cellular metabolism [43], the execution of apoptosis [6], [44], Ca^2+^ signaling [45], viral infection [46] and impact on various human pathologies [47]. However, the timing of the morphological changes experienced by mitochondria during mitosis in cells of mammals is unknown. In this regard, the generation of mammalian cell lines with green mitochondria allowed us to visualize *in vivo* the morphology and dynamics of mitochondria during mitosis (see supporting video S1 and Fig. S3) as well as within the context of elements of the cytoskeleton (Fig. 5).

The analysis of synchronized C9-pβGFP3′β cells at 4h after release from the metabolic block confirmed that a large proportion of the cells (>40%) were undergoing mitosis (Fig. 5). The morphology of mitochondria in cells in interphase is that of threads, or of a tubular network, that it is intertwined with the tubulin and actin cytoskeleton. Mitochondria in cells in interphase are preferentially clustered around the nucleus, although some mitochondria are also located in the cell periphery (Fig. 5). By the time cells enter mitosis the mitochondrial tubular network is disorganized and fission of the thread-like organelles had occurred with some of the mitochondria already appearing as punctuate organelles (especially in metaphase) (Fig. 5). During the early stages of mitosis mitochondria are excluded from the cellular space where the mitotic spindle is being assembled (prophase) as well as from the equator of the spindle where the chromosomes line up (metaphase) (Fig. 5). During anaphase mitochondria still display a punctuate morphology appearing evenly distributed in the cell periphery (Fig. 5). By the time of late-anaphase or early-telophase some punctuate mitochondria initiate the repopulation of the spindle equator after the segregation of the daughter chromosomes had occurred (Fig. 5). In late-telophase, just before cytokinesis, mitochondria initiate their change in morphology also appearing as thread-like structures (Fig. 5). Overall, the cellular distribution of mitochondria during mitosis appears not to be co-distributed with the majority of the tubulin and β-actin cytoskeleton (Fig. 5). Of note is the enrichment of β-actin in the cell cortex (the red layer beneath the plasma membrane) during mitosis (Figs. 5g, 5h) that governs the formation of filopodia (the thin red stiff protrusions on Fig. 5g) responsible for promoting the dramatic changes in cell shape during mitosis (see phase contrast images on supporting video S1 and Fig. S3).

## Discussion

The timing of the biosynthesis of mtDNA in cells of mammals during progression through the cell-cycle is nowadays a matter of debate. In this regard, the replication of mtDNA has been described to proceed continuously during the cycle [48], [49] or to occur at a specific stage of the cycle (late S-G2/M) after most nDNA synthesis has been completed [50], [51]. The lack of significant changes on the cellular representation of mtDNA in liver cells during cellular proliferation suggests that mtDNA and nDNA synthesis is coordinated and supports a concerted trans-activation of the replication of both genomes [52]. The synchronous replication of both genomes is not incompatible with localized [53] or delocalized synthesis of mtDNA [49] which might represent events of mtDNA turnover and/or repair of the nucleoid.

Consistent with the prominent role played by cardiolipin in translation of mitochondrial proteins and on the biogenesis of OXPHOS complexes ([54]–[56] and references therein) we observed that synthesis of the mitochondrial encoded COXI protein is fully accounted in S phase concurrently with the synthesis of cardiolipin and of the nuclear-encoded counterpart subunit COXIV. However, we observed that a fully developed ΔΨm is not attained until G2/M (Fig. 2D) [57]. The assembly and oligomerization of the H^+^-ATP synthase has been shown to play a critical role in the folding of the inner membrane into cristae morphology [58]–[60]. Moreover, in yeast cells the activity of the ATPase has been shown to be strictly required for the maintenance of mitochondrial integrity and mtDNA [61]. It is thus reasonable to suggest that the synthesis of β-F1-ATPase, and perhaps of other nuclear-encoded OXPHOS components whose synthesis is driven at G2/M, could rate-limit the development of a functional organelle during cellular proliferation.

The regulation of the expression of nuclear and mitochondrial genes during the cell cycle has been described at both the level of transcription [15], [62], [63] and by changes in the half-life of the mRNAs [15], [63]–[65]. However, the buildup of β-F1-ATPase during cellular proliferation occurs in the absence of changes in the cellular abundance of its mRNA consistent with the stringent translational control described for this transcript [21]–[25]. Moreover, the expression of a reporter driven from a chimeric mRNA containing the regulatory 3′UTR of β-mRNA recapitulated the same pattern and mechanism of expression of the endogenous protein during cellular proliferation, providing the first indication that translation at G2/M is also required for the synthesis of an essential component of mitochondria, and supporting that cell-cycle regulated changes in the translational efficiency of some OXPHOS transcripts play a prominent role in the biogenesis of mitochondria.

It is well established that *cap-*dependent translation is inhibited at mitosis as a result of multiple events that lead to the disruption of the eIF4F complex [66]. At this stage of the cycle *cap-*independent translation drives the synthesis of relevant cellular proteins from a set of internal ribosome entry site (IRES)-containing mRNAs [67]–[69]. Consistent with the synthesis of β-F1-ATPase at G2/M, the translation of β-mRNA has been shown to have a lesser dependence on eIF4E than other cellular mRNAs [32]. Moreover, the 3′UTR of β-mRNA acts as a translation enhancing sequence [24], [33] that displays IRES-like activity in dicistronics constructs [32]. Therefore, an important conclusion resulting from this work is that the 3′UTR-located IRES of β-mRNA is the target of cell cycle-dependent translational regulation.

The mammalian β-mRNA was the first nuclear-encoded transcript of the mitochondria shown to be localized and attached to the outer mitochondrial membrane [26]–[28]. Localization/translation of the mRNA requires two *cis*-acting elements respectively located in the ORF and in the 3′UTR of the mRNA [29]. The *cis*-acting element located on the ORF exerts a negative control on mRNA translation ([24] and Santamaria and Cuezva, unpublished observation). For efficient translation of β-mRNA it is required the IRES-like translation enhancing activity of the 3′UTR [24], [32]. Such translation enhancing activity of the 3′UTR of β-mRNA has also been observed in the 3′UTRs of other transcripts involved in the OXPHOS system of mammals [33] and yeast [31] cells. In fact, ablation of the 3′UTR in the yeast β-F1-ATPase gene resulted in cells with respiratory deficiency [30] although such phenotype has been attributed to a defective import of β-F1-ATPase rather than to the activity of the 3′UTR on translation. Consistent with this essential role of the 3′UTR of the transcript in the synthesis of β-F1-ATPase our efforts to develop a mouse with a genetic deletion on the 3′UTR of the β-F1-ATPase gene by homologous recombination (IngenKO, Australia) have resulted unsuccessful (data not shown).

The trans-acting factors of the 3′UTR of β-mRNA that have been shown to control the translation of the mRNA during liver development [24] and in oncogenesis [25] have poly A binding activity [70]. Unfortunately, the molecular nature of these RNA binding proteins still remains elusive. However, we have recently observed that both a reduced environment and a low ATP/ADP ratio favor the binding of these proteins to its target [70] what results in translation masking of the mRNA [22], [24], [25]. A burst of mitochondrial respiration has been noted upon serum stimulation of quiescent mammalian cells [9]. However, progression through the cell cycle is stringently controlled by the regulation of the metabolic pathways involved in energy generation [10], [11], [71]. Essentially, the “metabolic cycle” during cellular proliferation alternates between a first *oxidative phase* that is characterized by the biosynthesis of many cellular components (G1 phase) and is supported by the energy derived from mitochondrial activity followed by a *reductive phase* were the replication of DNA and the biosynthesis of mitochondria (S/G2/M phases) is supported by non-respiratory modes of energy generation [10], [11], [71]. The operation of the metabolic cycle in cells of mammals have received recent support after the observation that cyclin D1 represses mitochondrial function *in vivo* [13], [14]. We therefore suggest that control of β-mRNA translation in the reductive phase that prevails at G2/M should have additional players that control the interactions of trans-acting factors of the 3′UTR with its target to allow the translation of the mRNA in such unfavorable metabolic situation. Since the expression of β-F1-ATPase is down-regulated in most human cancers [38], [72] contributing to tumor progression [38], [39], [73] we believe that such regulators are responsible for promoting deregulated β-F1-ATPase expression in the cancer cell.

Finally, we presume that the rapid changes in mitochondrial morphology and dynamics that accompany mitosis might be of relevance for the appropriate segregation of the organelles and mtDNA into daughter cells during proliferation. We showed that fission of mitochondria is an early event of mitosis that might be triggered in response to the signaling cascades that accompany progression through the cell cycle. In fact, similar rapid fission events on the mitochondrial network are observed after treatment of the cells with an inhibitor of protein kinases [6]. Fission of the organelle is likely to be triggered by proteasomal degradation of mitofusins [74]. Functionally, fission of mitochondria assures a stochastic distribution of the organelles within the two daughter cells by a process that might be actively controlled and mediated by microtubules [75]. However, it has been reported that after mitochondrial fission 25–40% of the organelles lack mtDNA [76]. This situation could contribute to the asymmetric segregation of mtDNA into daughter cells explaining certain mtDNA depletion syndromes [47]. However, the fusion of mitochondria in late telophase, just before citokinesis, a process that is controlled by mitofusins and the development of ΔΨm [77], is likely to ameliorate the possible unequal distribution of mtDNA during cellular proliferation.

Overall, we have provided a comprehensive picture of the biogenesis of mammalian mitochondria during cellular proliferation and illustrated the relevance of translational control by the 3′UTR of an OXPHOS mRNA for the appropriate biogenesis of the organelle during cell cycle progression. We hope that these findings will contribute to the understanding of certain human pathologies that impinge on organelle malfunction as a result of deregulated cellular proliferation.

## Materials and Methods

### Cell cultures

Rat liver clone 9 (C9) and BHK (Baby Hamster Kidney) cells were grown at 37°C in DMEM with 10% fetal calf serum (FCS) [6]. Liver C9 cells were arrested at the beginning of S phase by a double thymidine block. In brief, subconfluent cultures were treated with 2 mM thymidine for 10 h. Afterwards, the cells were washed twice with DMEM and then grown for 6 hours in drug-free medium supplemented with 24 µM 2′-deoxycytidine. Cells were arrested for a second time with 2 mM thymidine for 10 h and after grown in DMEM containing 24 µM 2′-deoxycytidine.

### DNA constructs and cellular cloning

The pJMI-β-F1 plasmid [24], that contains the full-length rat liver β-F1-ATPase cDNA, was digested with Ban I for obtaining the fragment encoding the mitochondrial targeting sequence of β-F1-ATPase. The resulting cDNA fragment was cloned in the Eco 47 III site of plasmids CDL-GFP-β-3′UTR and CDL-GFP-Tfam-3′UTR [33], that express chimaeric RNAs of GFP with the 3′-UTRs of β-F1-ATPase and Tfam mRNAs, respectively. The sequence of the resulting plasmids pβ-GFP-β-3′UTR and pβ-GFP-Tfam-3′UTR, both expressing the same fusion protein between the mitochondrial targeting sequence of β-F1-ATPase and GFP (pβ-gfp), was verified by automated DNA sequencing. Cells were transfected at 60–80% confluence using 1 µl of FUGENE 6 (Boehringer Mannheim, Germany) and 0.5 µg of DNA/1×10^5^ cells in 1 ml of culture medium [33]. Positive cells expressing gfp in their mitochondria were selected by incubation with 40 µg/ml of geneticin (G-418) (GIBCO) and cloning cylinders (8×8 mm).

### Nucleic acids hybridizations

DNA and RNA were extracted from C9 cells and processed for hybridization of nucleic acids onto nylon membranes (GeneScreen) [33]. Membranes were incubated with [^32^P]dCTP-labeled DNA probes [25], exposed to X-ray films and analyzed by densitometry. Cytoplasmic RNA of sorted cells was extracted with RNeasy mini kit (Qiagen) following the manufacturer instructions. RNA quality was assessed previously to retro-transcription (RT) with High-Capacity cDNA Archive Kit (Applied Biosystems). qPCR was carried out on three different RT reactions. Oligonucleotides used for qPCR amplification were: 5′-gcaattattccccatgaacg-3′ and 5′-gggacttaatcaacgcaagc-3′ for 18S rRNA; 5′-ggtatggaatcctgtggcatccatgaaa-3′ and 5′-gtgctaaaacgcagctcagtaacagtcc-3′ for β-Actin mRNA and 5′-aaagctggtgcccctgaag-3′ and 5′-ggagatggtcatagtcacctgct-3′ for β-F1-ATPase mRNA. For qPCR, 5 ng of cDNA (template) and 0.5 µM of each primer together with Power Sybr Green PCR Master Mix (Applied Biosystems) were used following the protocol: 10′×95°C followed by 20–40 cycles of denaturation (15″×95°C) and annealing-elongation (1′×60°C) with fluorescence acquisition at 60°C. A melting curve (15″×95°C, 15″×60°C and 15″×95°C) with fluorescence acquisition at 60° to 95°C was included in each qPCR. The amplification efficiency of each primer was empirically determined and applied to the relative quantification of the data using qbase software (http://medgen.ugent.be/qbase/).

### Western-blots

Cells were recovered from the plates by trypsin treatment, washed twice with PBS and boiled in loading buffer. Cellular proteins were fractionated on SDS-12% PAGE and then transferred onto PVDF membranes for immunoblot analysis [25]. The primary monoclonal antibodies used were: anti-GFP (Clontech, 1∶1000), anti-α-tubulin (Sigma, 1∶1000), anti-hsp60 (Stressgene SPA807, 1∶3000), anti-COXIV (Molecular Probes, 1∶100) and anti-cyclin B1 (Santa Cruz Biotechnology, 1∶100). The primary polyclonal antibody used was anti-β-F1-ATPase (1∶30000) [38]. Peroxidase-conjugated anti-mouse or anti-rabbit IgGs (Nordic Immunology, 1∶3000) were used as secondary antibodies. The blots were developed using the ECL® reagent (Amersham Pharmacia Biotech, Little Chalfont, U.K.).

### Mitochondrial membrane potential (ΔΨm) and mitochondrial mass

The fluorescent TMRM^+^ and NAO probes (Molecular Probes, Eugene, Oregon) were used to analyze ΔΨm and mitochondrial mass by flow cytometry, respectively [6]. The cellular fluorescence intensity was measured using a FACScan flow cytometer (Becton-Dickinson, San José, CA.). For each analysis 10,000 events were recorded.

### Flow Cytometry Analysis

For the determination of the cellular content of mitochondrial proteins in the different phases of the cell cycle, ∼1×10^6^ C9 cells were washed in ice-cold staining buffer (1% BSA, 1% FCS and 0.01% sodium azide in PBS) and further incubated with primary monoclonal antibodies against β-F1-ATPase (Molecular Probes, 0.02 µg/µl), Hsp60 (Stressgene, 0.01 µg/µl), COXI and COXIV (Molecular Probes, both at 0.04 µg/µl) in staining buffer supplemented with 0.3% saponin. The cells were then washed in ice-cold PBS-saponin buffer and incubated in the dark for 20 min with goat anti-mouse IgG conjugated to Alexa 488. Finally, the cells were resuspended in PBS-staining buffer containing 5 µg/ml of propidium iodide (Sigma) and 100 µg/ml RNase A and further incubated for 20 min at 37°C.

For the determination of the cellular content of the expressed gfp in C9-pβGFP3′β and C9-pβGFP3′Tfam cells, ∼1×10^6^ cells were fixed with 0.25% paraformaldehyde in PBS for 5 min. After, the cells were resuspended in 70% ice-cold ethanol, collected by centrifugation and finally incubated for 30 min at 37°C in PBS containing 100 µg/ml of RNase A and 20 µg/ml of propidium iodide. The mean fluorescence intensity of the cells was determined after excitation at a wavelength of 488nm on a FACScan. For computer analysis only the signals from single cells were considered (10,000 cells/assay). Data analysis was carried out using Flow Jo 6.4.1 for Mac.

For the separation of the cells in G0/G1, S and G2/M phases of the cycle, cells were stained for 10 minutes with 5 µg/ml of Hoescht 33342 added to the culture media. Cells (∼5×10^6^ cells/ml) were resuspended in PBS containing 5 mM EDTA, 25 mM HEPES pH 7.0 and the cellular aggregates removed by filtration through 70 µm pore filters. Cells were sorted in a FACSCVantage SE (BD Biosciences, Erembodegen, Belgium) according to their DNA content and collected in 0.5 ml of FCS. The purity of each fraction was assessed in an aliquot of propidium iodide stained cells.

### Immunofluorescence/confocal microscopy

Fluorescence and indirect immunofluorescence microscopy was performed on mitochondria-tagged gfp C9 cells as recently described in detail [6]. The primary antibodies used were mouse monoclonal anti-α-tubulin and anti-β-actin (both from Sigma, 1∶200). The primary polyclonal antibody used was anti-β-F1-ATPase (1∶1000) [38]. After three PBS rinses, the cells were incubated for 1 h in the dark with goat anti-mouse or goat anti-rabbit IgGs conjugated to Alexa 594 (Molecular Probes) at 1∶1000 dilution. The nuclei were stained with ToPro3 (Molecular Probes). Cellular fluorescence was analyzed by confocal microscopy using a Biorad Radiance 2000 Zeiss Axiovert S100TV using the following excitation/emission wave lengths: green (498/516 nm), red (590/617 nm) and blue (642/661 nm). The fluorescence emission of gfp was also analyzed in a Leica DMIRB fluorescence microscope equipped with a gfp excitation filter (Leica, BP 470/40).

### Immunocytochemical localization of gfp by electron microscopy

Cells were fixed in freshly prepared 4% paraformaldehyde in 0.1 M Sörensen phosphate buffer, pH 7.2, for 2 h at 4°C. The free-aldehyde groups were quenched with 50 mM ammonium chloride in PBS for 60 min at 4°C. Samples were dehydrated in acetone and finally processed for embedding in Lowicryl K4M (Polysciences Europe, Eppelheim, Germany) [27]. Ultrathin sections were collected in collodion/carbon-coated nickel grids. Grids were incubated for 5 min with PBS containing 1% BSA and then incubated with anti-gfp (1∶100). After three washes with PBS, grids were incubated for 45 min with goat anti-rabbit IgGs conjugated with 10 nm colloidal gold (British BioCell, Cardiff, UK). The grids were washed twice in PBS and distilled water, and air-dried. Counterstaining was performed with 2% aqueous uranyl acetate (6 min) and Reynolds lead citrate (45 s). Observation was performed in a Jeol 1010 electron microscope under 80 kV accelerating voltage.

### Online supplementary material

For live imaging presented in the supplementary video, mitochondria-tagged gfp C9 cells were grown on glass plates and live images were taken in a Cell Observer Zeiss equipment using a Zeiss Axiovert 200 inverted microscope equipped with a Coolsnap FX CCD camera (Roper Scientific). Phase contrast and fluorescence images were taken every 3 min for 20 frames (1 h) with a 40× objective and a gfp filter. The Metamorph 6.1r6 (Universal Imaging) program was used for image processing.

## Supporting Information

Figure S1 Analysis of the level of synchrony in cell cultures. C9 cells were treated as described to induce the arrest of the cell cycle at the G0/G1-S transition. After the release from the arrest, cells were grown and analyzed by flow cytometry at different time-points after DNA staining with propidium iodide. The panels illustrate the cell cycle profiles of representative experiments for control, arrested and 2-h and 4-h after the release from the arrest. The graph summarizes the analysis of cell distribution during the cell cycle of four independent synchronization experiments. Open squares, control non-arrested cells; closed circles, arrested cells and closed triangles cells at 4 h after release from the arrest. The results shown are the means±S.E.M. * and †, P<0.01 when compared with control and arrested cells, respectively.
(0.17 MB TIF)

Figure S2 Accumulation of mitochondrial proteins during the cell cycle. Determination of the expression level of mitochondrial proteins was carried out by flow cytometry after the staining of the cells with specific antibodies against β-F1-ATPase, Hsp60, COXI and COXIV. Irrelevant isotype-specific IgGs and antibodies against the T-cell receptor complex (TCRVβ 8) were used as controls. The histograms in (a) show the overlay of the fluorescence intensity of each of the primary antibodies used (in blue) with the signal provided by the secondary antibody (in red) for the entire cell population analyzed. The histograms in (b) show the fluorescence intensity of the cells in G0/G1 (in red), S (in blue) and G2/M (in green) phases of the cell cycle. The mean fluorescence intensity for each cellular subpopulation is shown. Representative histograms are shown for each of the conditions tested. The results illustrate the lack of overlapping of the fluorescent signal of the mitochondrial markers with that of the secondary antibody or of the two controls (IgGs and TCRVβ 8).
(0.17 MB TIF)

Figure S3 Changes in cellular morphology and on the mitochondrial network during mitosis. Different time-frames of phase contrast (left panel) and fluorescence (right panel) images taken from the supporting video 1 illustrate the changes in cellular morphology and of the mitochondrial network during mitosis, respectively. a, interphase; b, prophase; c, prometaphase; d, metaphase; e, anaphase and f, telophase. Phase contrast images allowed the visualization of filopodia (b,c) and chromosomes (d,e,f).
(0.65 MB TIF)

Video S1 Changes in cellular morphology and on the mitochondrial network during mitosis. Phase contrast (left panel) and fluorescence (right panel) images from mitochondria-tagged gfp C9 cells were obtained every 3 min. The cellular shape changes dramatically during mitosis. In prophase/prometaphase, the plasma membrane promotes the protrusion of thin filopodia for cellular detachment triggering the clustering of mitochondria towards the cell nuclei. Fission of thread-like mitochondria is observed as a very early event of the initiation of mitosis (∼12–15 min) and is clearly finalized in metaphase. Some images representing individual frames of the movie are shown in Fig. S3.
(5.04 MB AVI)
