# Supplementary figures and images for: Biogenesis and Dynamics of Mitochondria during the Cell Cycle: Significance of 3′UTRs
Source: PLoS One. 2006 Dec 20;1(1):e107. doi: 10.1371/journal.pone.0000107 (PMC1762426; doi:10.1371/journal.pone.0000107)

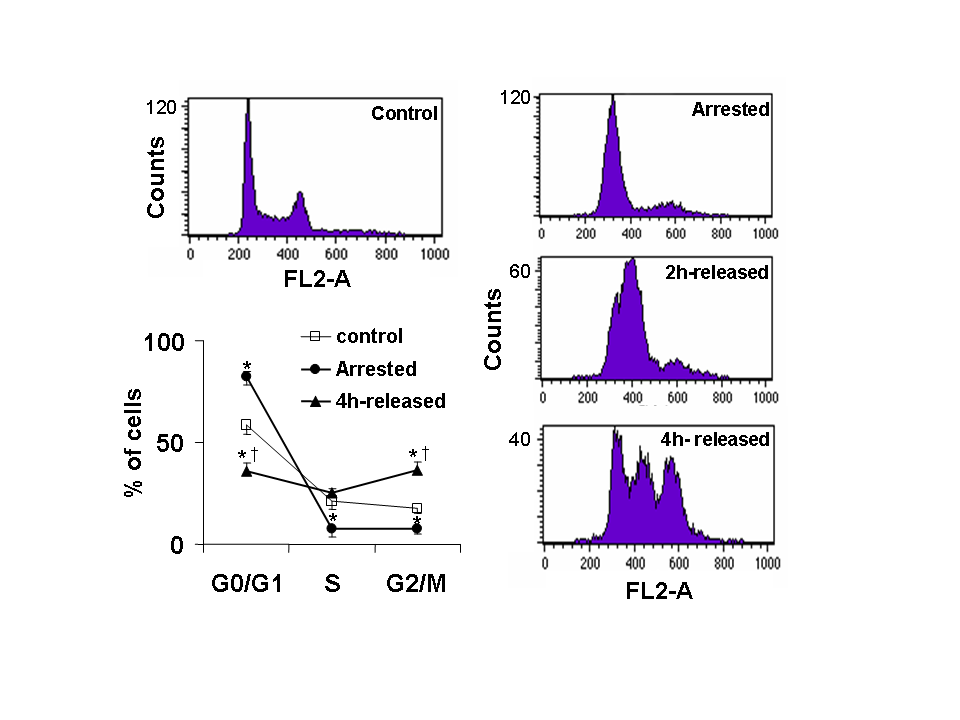

Supplement: Figure S1 — Analysis of the level of synchrony in cell cultures. C9 cells were treated as described to induce the arrest of the cell cycle at the G0/G1-S transition. After the release from the arrest, cells were grown and analyzed by flow cytometry at different time-points after DNA staining with propidium iodide. The panels illustrate the cell cycle profiles of representative experiments for control, arrested and 2-h and 4-h after the release from the arrest. The graph summarizes the analysis of cell distribution during the cell cycle of four independent synchronization experiments. Open squares, control non-arrested cells; closed circles, arrested cells and closed triangles cells at 4 h after release from the arrest. The results shown are the means±S.E.M. * and †, P<0.01 when compared with control and arrested cells, respectively. (0.17 MB TIF) [file pone.0000107.s001.tif]

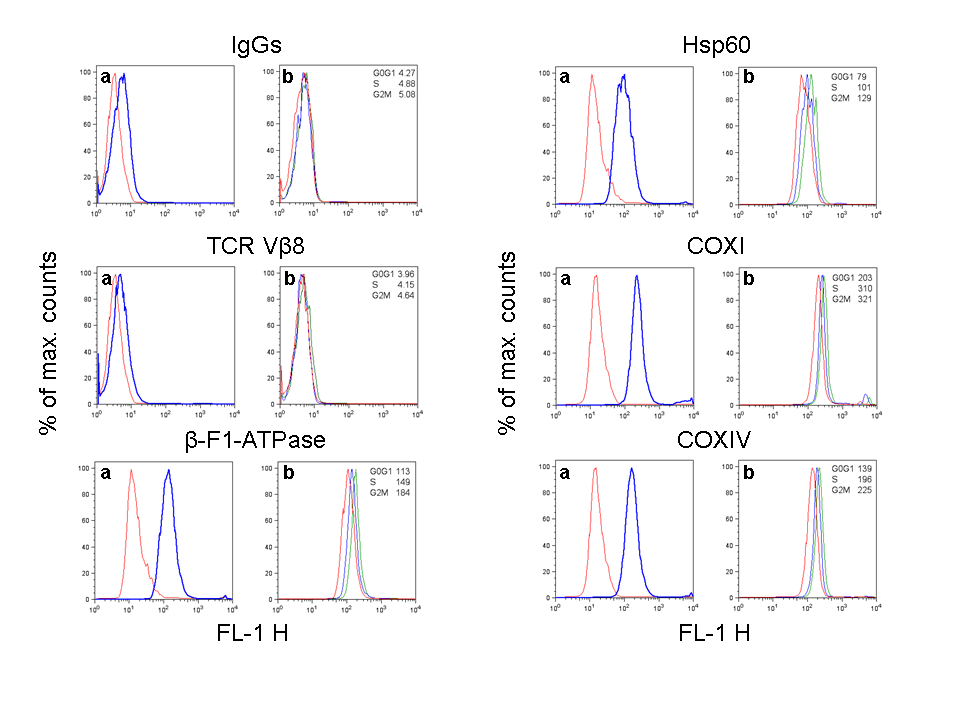

Supplement: Figure S2 — Accumulation of mitochondrial proteins during the cell cycle. Determination of the expression level of mitochondrial proteins was carried out by flow cytometry after the staining of the cells with specific antibodies against β-F1-ATPase, Hsp60, COXI and COXIV. Irrelevant isotype-specific IgGs and antibodies against the T-cell receptor complex (TCRVβ 8) were used as controls. The histograms in (a) show the overlay of the fluorescence intensity of each of the primary antibodies used (in blue) with the signal provided by the secondary antibody (in red) for the entire cell population analyzed. The histograms in (b) show the fluorescence intensity of the cells in G0/G1 (in red), S (in blue) and G2/M (in green) phases of the cell cycle. The mean fluorescence intensity for each cellular subpopulation is shown. Representative histograms are shown for each of the conditions tested. The results illustrate the lack of overlapping of the fluorescent signal of the mitochondrial markers with that of the secondary antibody or of the two controls (IgGs and TCRVβ 8). (0.17 MB TIF) [file pone.0000107.s002.tif]

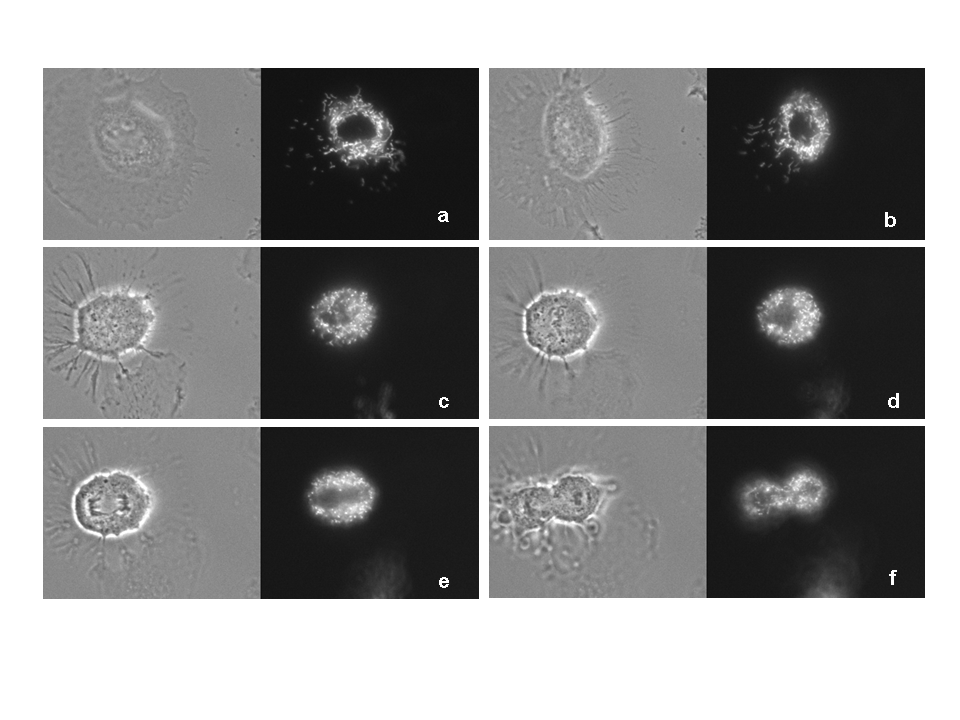

Supplement: Figure S3 — Changes in cellular morphology and on the mitochondrial network during mitosis. Different time-frames of phase contrast (left panel) and fluorescence (right panel) images taken from the supporting video 1 illustrate the changes in cellular morphology and of the mitochondrial network during mitosis, respectively. a, interphase; b, prophase; c, prometaphase; d, metaphase; e, anaphase and f, telophase. Phase contrast images allowed the visualization of filopodia (b,c) and chromosomes (d,e,f). (0.65 MB TIF) [file pone.0000107.s003.tif]
